# Pelvic Tilt Increases the Risk of Impingement and Alters Impingement Type in Total Hip Arthroplasty: A Patient‐Specific Simulation Study

**DOI:** 10.1002/jor.26085

**Published:** 2025-04-12

**Authors:** Arnab Palit, Mark A. Williams, Vineet Seemala, Mike Donnelly, Tobias Renkawitz, Markus Weber

**Affiliations:** ^1^ WMG The University of Warwick Coventry UK; ^2^ Department of Orthopaedic Surgery Regensburg University Regensburg Bad Abbach Germany; ^3^ Department of Orthopedic and Trauma Surgery Barmherzige Brueder Regensburg Regensburg Bad Abbach Germany

**Keywords:** impingement, pelvic tilt, randomized control trial (RCT), range of motion (ROM) simulation, total hip arthroplasty (THA)

## Abstract

Impingement is a significant complication following total hip arthroplasty (THA), resulting in restricted range of motion (ROM). Pelvic tilt (PT) and its variation could alter both post‐THA ROM and impingement types, which remains relatively unexplored in the literature. Therefore, this study aims to investigate the impact of PT changes on post‐THA ROM and impingement types. Subject‐specific ROM was simulated using 3D‐CT and clinical data for 56 THA patients. Subsequently, the effect of no‐PT, standing preoperative (StPT_0_) and postoperative PT at 6 (StPT_6_) and 12 months (StPT_12_) on maximum ROM (flexion, extension, abduction, adduction, external rotation (ER) and internal rotation at 90° Flexion (IR@90°Flex)) and impingement types (implant‐to‐implant impingement (ITII), implant‐to‐bone impingement (ITBI), and bone‐to‐bone impingement (BTBI)) were investigated. Stong correlations existed between PT and flexion (*R* ^2^ ≥ 0.686), extension (*R* ^2^ ≥ 0.527), and IR@90°Flex (*R* ^2^ ≥ 0.547). Anterior PT exceeding 8.1° and 11.8° were linked to decreased flexion below 110° and IR@90°Flex below 30°, respectively. Each 10° increase in anterior PT resulted in a 10° reduction in flexion and a 10.7° reduction in IR@90°Flex. Impingement types due to PT remained unchanged for flexion/extension, with increased ITII for abduction (8.9%), adduction (23.2%), and IR@90°Flex (16.1%), and increased BTBI (16.1%) for ER. In total, 12.5% and 19.6% of patients experienced clinically relevant ROM change for flexion and IR@90°Flex, respectively for StPT_0_–StPT_6_. However, it affected below 5.4% cases when comparing StPT_6_ and StPT_12_. Minor changes in impingement type (< 6% of cases) were observed due to changes in PT before and after THA, as well as temporal changes in PT post‐THA. However, PT had a substantial impact on impingement types when comparing ROM without considering PT to ROM with PT included. Specifically, anterior PT was associated with reduced flexion and IR@90°Flex, indicating a higher risk of impingement. PT changes over time may lead to clinically relevant alterations in ROM but not impingement types.

**Trial Registration:** German Clinical Trials Register; Main ID: DRKS00000739.

## 1 Introduction

Impingement after total hip arthroplasty (THA) may lead to restricted range of motion (ROM), diminishing hip functionalities, and heightening pain [1, 2]. Motions beyond the impingement point result in femoral head dislocation, a major cause of revision surgery [3, 4, 5], with ~90% of dislocations attributed to impingement [6]. Enhanced post‐THA ROM can mitigate impingement, reducing the risk of dislocation during activities of daily living (ADL) [7]. On the other hand, pelvis significantly rotates in sagittal plane during ADLs [8]. The amount of PT as well as the direction of rotation are substantially different amongst various functional hip joint motions, and these variations are also highly patients‐specific [8, 9, 10, 11]. Additionally, the average change in PT following THA has been documented as minor, but the individualized change for each patient could be significantly greater [12, 13, 14]. A change in postoperative PT of 5° could result in considerable changes to the specific safe zone for the orientation of the acetabular component based on patient's anatomy [14, 15]. Consequently, the alteration in PT after THA could impact both the ROM and the likelihood of impingement. Furthermore, it is uncertain whether changes in PT, including both magnitude and direction, could shift impingement type such as implant‐to‐implant impingement (ITII), implant‐to‐bone impingement (ITBI), and bone‐to‐bone impingement (BTBI) [16, 17, 18, 19, 20]. Despite significant research on post‐THA PT [21] and ROM [22, 23], the impact of PT on post‐THA ROM, including maximum ROM and impingement type, remains inadequately explored.

Therefore, the aim of this study was to investigate the effect of PT and its changes on post‐THA maximum ROM and impingement type. PT was captured pre‐THA (StPT_0_) and post‐THA at 6 (StPT_6_) and 12 months (StPT_12_). The investigation utilized subject‐specific hip motion simulation using 3D‐CT and clinical data of 56 patients after THA. The impingement types examined were ITII, implant‐to‐bone impingement (ITBI), and bone‐to‐bone impingement (BTBI).

## 2 Methods

**Figure 1:**
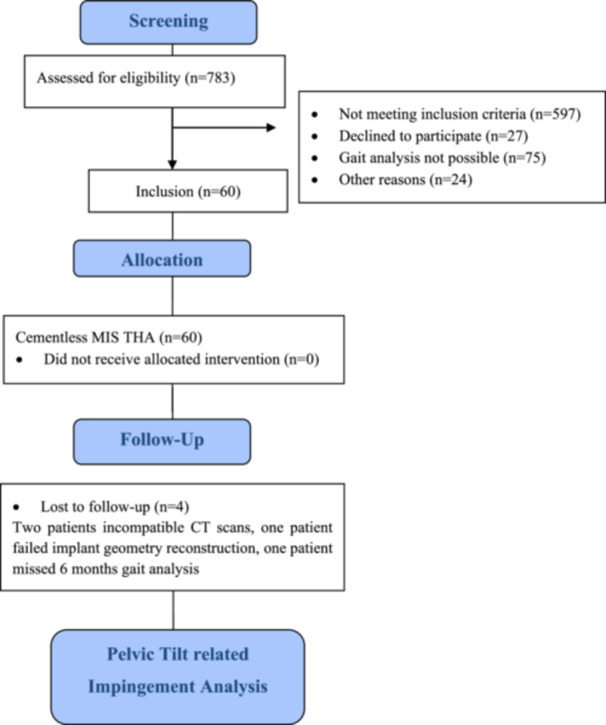
Flow diagram of the participants in the study.

The study utilized data from an observer‐blinded randomized controlled trial (RCT) approved by the local Medical Ethics Committee (10‐121‐0263). This study is a secondary retrospective data analysis. Approval was obtained for CT scans of the pelvic and femur, essential for evaluating postoperative conditions ~6 weeks postsurgery. The RCT was registered in the German Clinical Trials Register under Main ID DRKS00000739. The study design details, including sample size, inclusion and exclusion criteria, randomization, patient recruitment, and surgical procedures, are provided in references [24, 25], while the flow chart of the cohort is shown in Figure 1.

From the primary study cohort, a subgroup of 60 participants from the gait lab was selected. Four patients were excluded from analysis: one missed the 6‐month follow‐up, one had failed implant geometry construction due to CT scan quality, and two had incompatible CT data sets for impingement analysis (Figure 1). The final analysis included 56 data sets, with patient characteristics and clinical data. This current study, an independent secondary outcome analysis, obtained approval from the Biomedical and Scientific Research Ethics Committee (BSREC) at the University of Warwick, UK (ref: REGO‐2018‐2229 AM01 and BSREC 66/22‐23) to conduct simulation analysis on these data.

### 2.1 Patient Specific Clinical Data

Clinical and radiographic data were provided by the Department of Orthopaedic Surgery, Regensburg University Medical Centre. This included patient‐specific pelvis, femur, and implant geometries derived from 3D‐CT scans (Somatom Sensation 16; Siemens, Erlangen, Germany) of the hip, alongside details of implant type, size and achieved cup/liner inclination and anteversion angles with respect to anterior pelvic plane (APP). Press‐fit acetabular components (Pinnacle cup, DePuy, Warsaw, IN, USA) and cementless hydroxyapatite‐coated stems (Corail stem, DePuy, Warsaw, IN, USA) with metal heads of 32 mm were used universally for THA. The patients underwent minimally invasive THA using an anterolateral approach, followed by a clinical CT scan 6 weeks after surgery. The study included two functional post‐THA pelvic tilt measurements obtained from gait lab experiments: standing pelvic tilt at 6 months (StPT_6_) and at 12 months postsurgery (StPT_12_), as well as a functional pre‐THA measurement (StPT_0_). The functional pelvic tilt was measured from the gait lab measurements analyzed with the Anybody Modeling System and 3D‐CT fusion, following the method outlined by Weber et al. [26]. All the pelvic tilts were measured with respect to the APP, with all other components set to 0.

### 2.2 Construction of Bone and Implant Geometries

**Figure 2:**
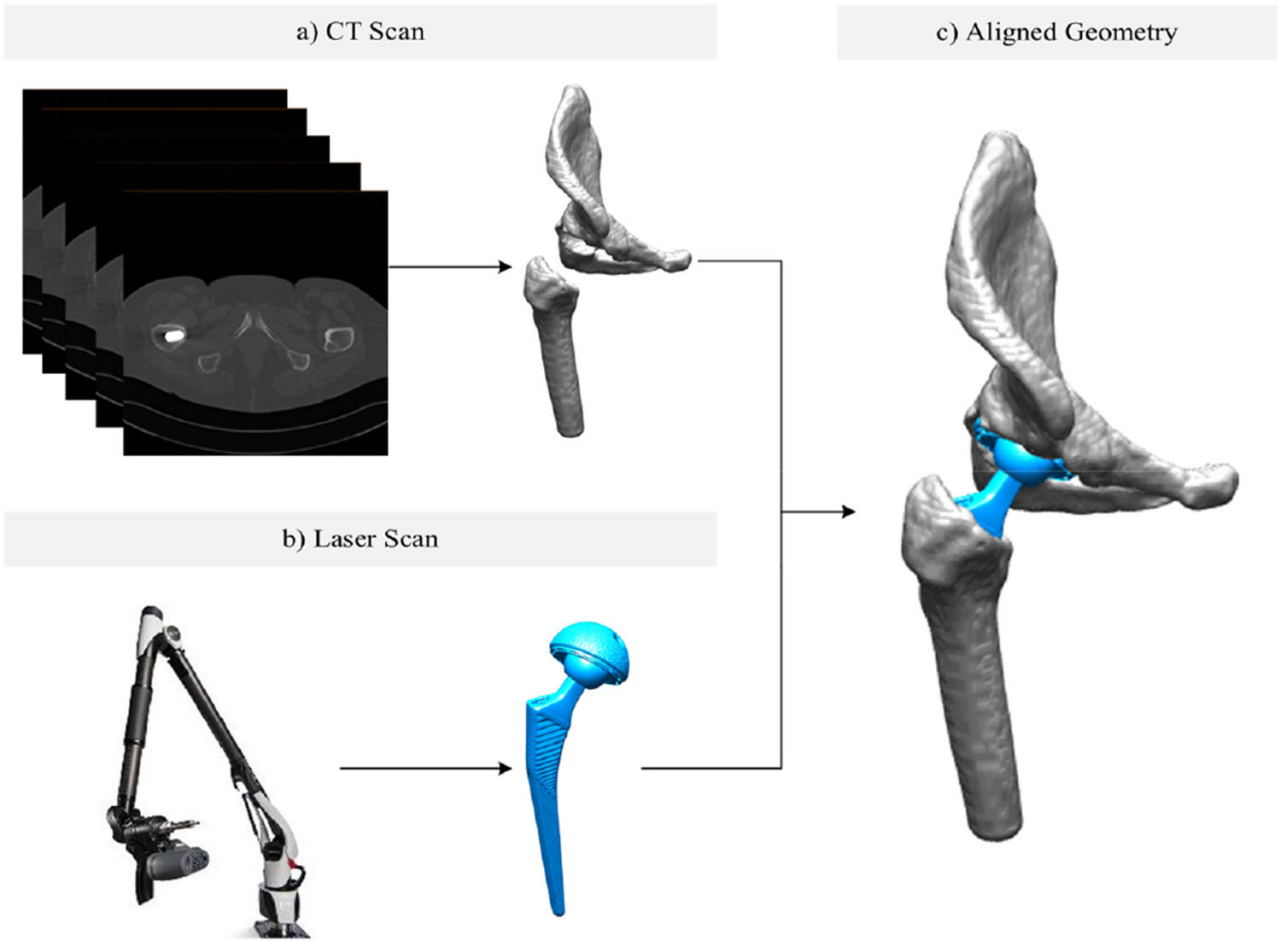
Hip joint simulation input geometry: (a) pelvis and femur geometry from the CT scan, (b) implant geometry from the laser scan, and (c) bone and implant geometry post alignment.

Manual CT segmentation was performed on the pelvis and femur, as well as on the metal acetabular and femoral implant components, by an independent external institute (Fraunhofer MEVIS, Bremen, Germany), which was blinded to individual patient data. Cup inclination, anteversion, and stem anteversion were assessed by the independent external institute using image‐processing software (based on MeVisLab, MeVis, Bremen, Germany), as previously outlined [25]. Implant geometries for 56 patients (Table 1) were reconstructed via laser scanning at WMG, University of Warwick, using a Nikon ModelMaker H120 scan head mounted on a portable scan arm MCAx25+ (Figure 2b). Data were collected in Nikon's proprietary software Focus. The scan head employs blue‐light laser technology with a minimum resolution of 35 µm and a combined accuracy with the scanning arm of 32 µm (2σ). The constructed implant geometries were then digitally placed onto the native bone geometry to replicate the THA implant positioning achieved during the surgical procedure, as described in the subsequent section.

**Table 1:** Patient characteristics and clinical data.

Patient characteristic		
Sex (female/male)		32/24
Age		62.6 ± 7.9
BMI		27.0 ± 4.0
Treatment side (left/right)		29/27
Kellgren score		8 (6–10)

Implant information		
Cup	Size	52 (48–62)
	Inclination (°)	42.5 ± 5.2
	Anteversion (°)	18.3 ± 6.9
	Coverage (%)	87.4 ± 9.0
Stem	Size	12 (9–16)
	Standard (%)/high offset (%)	39.3%/60.7%
	Anteversion (°)	9.1 ± 10.4

### 2.3 Implant Positioning on Native Bone Geometries

Initially, patient‐specific laser‐scanned cup and liner geometries were aligned by placing their centers at the origin and aligning the implant axis with the *z*‐axis using Geomagic 2014 (3D Systems, Morrisville, North Carolina). Then, each patient's specific cup and liner were aligned with the pelvis, translating their centers to the hip joint center (HJC) and rotating the implant axis based on the patient's inclination and anteversion angles from surgery. The head and stem were aligned based on each patient's head offset data, and insertion depth was calculated to ensure proper contact between the head slot and stem without interference. The femoral component from the laser scan was aligned with the femoral component from the CT scan using best‐fit registration in Geomagic. Figure 2c illustrates the aligned bone and implant geometry, combining the bone geometry from the CT scan and the implant geometry from the laser scan. These aligned geometries were utilized to simulate hip joint motion.

### 2.4 Hip ROM Simulation and Impingement Detection

**Figure 3:**
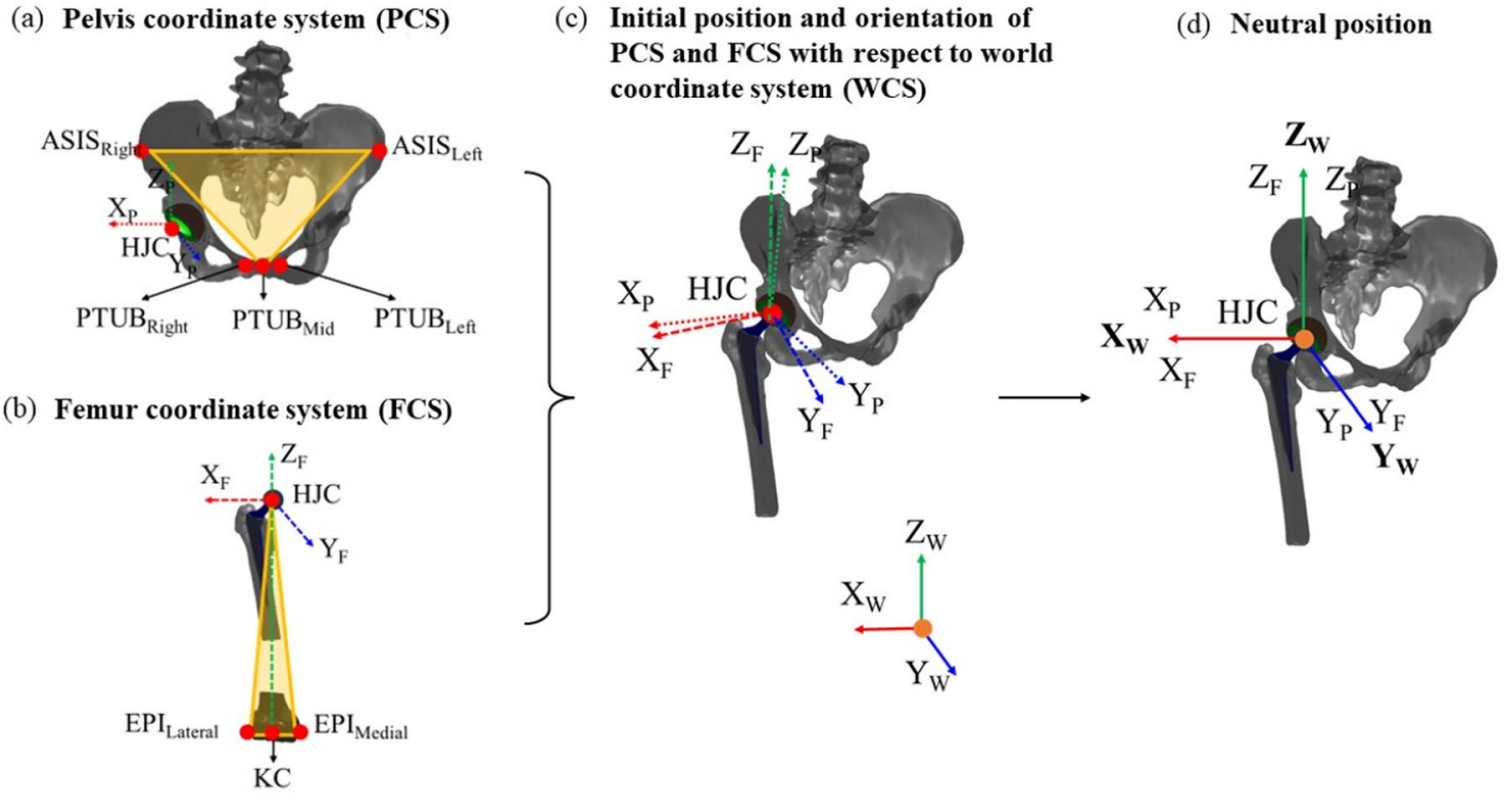
Definition and alignment of PCS and FCS based on landmarks to WCS: (a) pelvic landmarks (ASIS_Right_, ASIS_Left_, PTUB_Right_, PTUB_Left_) form PCS (*X*_P_, *Y*_P_, *Z*_P_) with HJC as origin; (b) femoral landmarks (EPI_Lateral_, EPI_Medial_), Knee Center define FCS (*X*_F_, *Y*_F_, *Z*_F_) with HJC as origin; (c) initial misalignment of PCS and FCS with WCS (*X*_W_, *Y*_W_, *Z*_W_); and (d) alignment of PCS and FCS with WCS to establish neutral pelvis and femur position.

Both the pelvic coordinate system (PCS) and femoral coordinate system (FCS) were established using the four pelvic and three femoral landmarks, respectively (Figure 3a), following the ISB recommendations [27]. The PCS and FCS were aligned to mimic the neutral position by first aligning the origins of the PCS and FCS with the origin of the world coordinate system (WCS). Subsequently, the PCS and FCS were rotated so that their three coordinate axes coincided with the world coordinate axes as depicted in Figure 3. The construction and alignment of the PCS and FCS were detailed in authors' previous work [28].

First, the subject‐specific PT was implemented at neutral position by rotating the pelvis around world *x*‐axis (*X*_W_) using *R*_*x*_ where angle *α* is the PT (Equation 1). Following the PT of pelvis, the femoral (with implant) movement was implemented as follows: (a) *R*_*x*_ represented the rotation with respect to world *x*‐axis (*X*_W_) to generate flexion–extension movement of angle *α*, (b) *R*_*y*_ depicted the rotation around global *y*‐axis (*Y*_W_) to generate abduction–adduction of angle *β*, and (c) *R*_*z*_ created the rotation around global *z*‐axis (*Z*_W_) to generate internal–external rotation of angle *γ*.

(1) $$\begin{array}{l} R_{x}=\left[\begin{array}{lcc} 1 & 0 & 0\\ 0 & \cos\alpha & -\sin\alpha \\ 0 & \sin\alpha & \cos\alpha \end{array}\right];\;R_{y}=\left[\begin{array}{ccc} \cos\beta & 0 & \sin\beta \\ 0 & 1 & 0\\ -\sin\beta & 0 & \cos\beta \end{array}\right];\\ \;R_{z}=\left[\begin{array}{ccc} \cos\gamma & -\sin\gamma & 0\\ \sin\gamma & \cos\gamma & 0\\ 0 & 0 & 1 \end{array}\right]. \end{array}$$

For the computation of the impingement, a collision check function was executed within Matlab 2021b (The MathWorks Inc., Natick, MA), utilizing the MEX API interface. This function was created by utilizing the “collision detection” algorithm supplied by the Proximity Query Package (PQP) library [28]. The femur with implant systematically moved by 0.5° increments until impingement was detected to calculate the limiting ROM. It should be noted that the effect of soft tissue has not been taken into account in the hip ROM simulation.

### 2.5 Study Design and Statistical Analysis

In this study, six hip joint motions were considered for calculating the limiting ROM: (a) Flexion (Flex), (b) Extension (Ext), (c) Abduction (Abd), (d) Adduction (Add), (e) External rotation (ER) at neutral femur position, and (f) Internal rotation (IR) at 90° flexion (IR@90°Flex). Impingement types were recorded during ROM calculations. Generalized post‐THA reference values for ADLs were adopted from previous studies [28, 29, 30, 31, 32], including 110° flexion, 30° extension, 45° ER at 0° hip flexion, 30° internal rotation at 90° hip flexion, 50° abduction, and 30° adduction. These values served as benchmarks to assess the influence of PT on ROM changes. The clinical relevance of ROM changes due to PT were categorized as follows: (a) low for differences < 10°, (b) medium for differences ranging from 10° to 20°, and (c) high for differences exceeding 20°. The hip ROM was calculated at four different PT values: (i) no PT, (ii) StPT_0_, (iii) StPT_6_, and (iv) StPT_12_. An alpha level (*α*) of 0.05 was used for all statistical *t*‐tests.

## 3 Results

### 3.1 Pelvic Tilt Distribution

**Figure 4:**
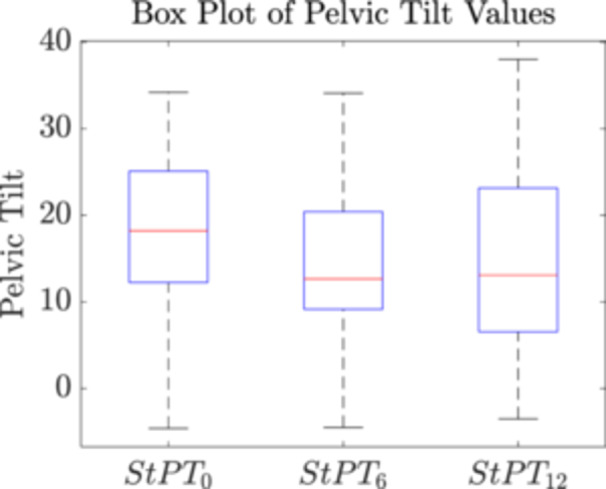
Standing pelvic tilt distribution measured at three time points: prior to surgery and at 6 and 12 months after THA.

Figure 4 presents a box plot illustrating the distribution of pelvic tilt measured at different time points. The mean ± standard deviation (SD) of standing PT was 18.9° ± 8.8° before surgery (StPT_0_), 14.1° ± 8.6° at 6 months post‐THA (StPT_6_), and 14.7° ± 10.4° at 12 months post‐THA (StPT_12_). The average values were similar, but patient‐specific PT varied by a significant amount. There was a significant decrease in pelvic tilt between the StPT_0_ (18.87 ± 8.76) and StPT_6_ (14.08 ± 8.66), *t*(55) = 5.93, *p* = 0.004. Similarly, a significant decrease was observed between the StPT_0_ (18.87 ± 8.76) and StPT_12_ (14.68 ± 10.44), *t*(55) = 4.28, *p* = 0.023. However, post‐THA, pelvic tilt remained relatively stable, as no significant difference was found between the StPT_6_ (14.08 ± 8.66) and StPT_12_ (14.68 ± 10.44), *t*(55) = −0.64, *p* = 0.744. Despite the lack of a statistically significant change in the population average between StPT_6_ and StPT_12_, individual patient‐specific changes in pelvic tilt may still be considerable.

### 3.2 Correlation of PT and Maximum ROM Within the Study Cohort

**Figure 5:**
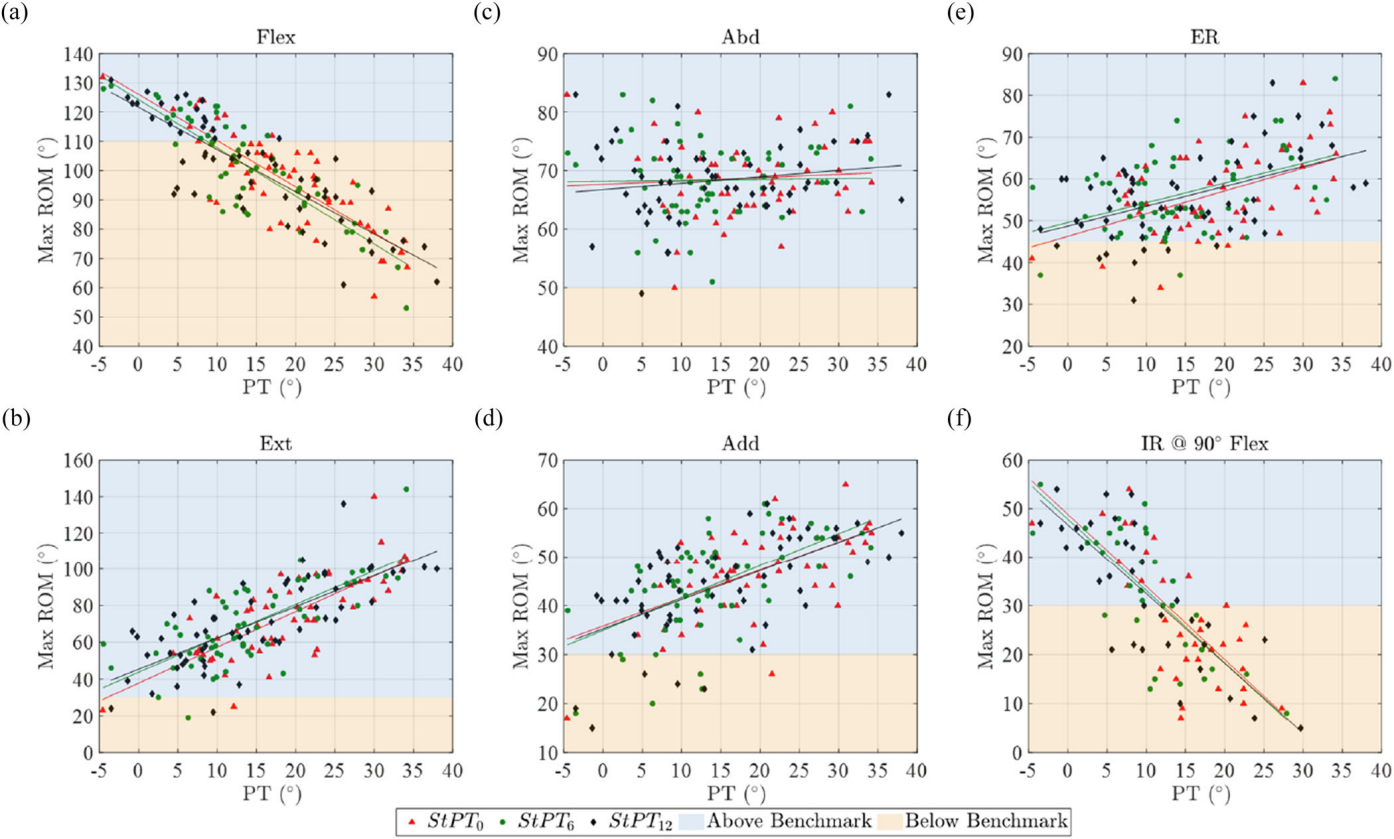
Maximum ROM for six hip motions with three standing pelvic tilts (StPT_0_, StPT_6_, and StPT_12_). (a) Flexion (Flex), (b) Extension (Extn), (c) Abduction (Abd), (d) Adduction (Add), (e) External Rotation (ER) at femur's neutral position (no Flex/Extn and no Abd/Add), and (f) Internal Rotation (IR) at 90° flexion with no Abd/Add. The maximum ROM values corresponding to StPT_0_, StPT_6_, and StPT_12_ are represented by red triangles, green circles, and black diamonds, respectively. The ROM above the reference benchmark (see Section 2.5) values is shaded in blue, and the ROM below the benchmark is shaded in yellow.

The ROM simulation revealed a strong correlation between maximum flexion (*R* ^2^ ≥ 0.686), extension (*R* ^2^ ≥ 0.527), and internal rotation at 90° of flexion (*R* ^2^ ≥ 0.547) and the PT in the standing position measured at the three different time points. However, no clinically relevant correlation was found for ER, abduction, and adduction (Table 2). Based on these regression fittings, it was observed that an anterior PT of 8.1° or more was associated with reduced hip joint flexion below the reference value of 110°. Similarly, an anterior PT of 11.8° or more correlated with reduced IR at 90°of flexion below the required benchmark of 30° (Figure 5). On the other hand, the ROM was consistently above the reference values for most of the study cohort for extension (> 30°), abduction (> 50°), adduction (> 30°), and ER (> 45°) (Figure 3).

**Table 2:** Linear fit statistics for ROM and PT correlations: (a) coefficient of determination (*R* ^2^), (b) root mean squared error (RMSE).

ROM	PT	*R* ^2^	RMSE	ROM values Mean ± SD (°)
Flexion	StPT_0_	0.68	9.4	96.3 ± 16.7
	StPT_6_	0.71	9.1	101.1 ± 16.9
	StPT_12_	0.712	9.6	100.4 ± 17.9
Extension	StPT_0_	0.576	14.7	74.5 ± 22.4
	StPT_6_	0.527	15.2	69.7 ± 22.0
	StPT_12_	0.579	15.2	70.3 ± 23.3
Abduction	StPT_0_	0.006	6.4	68.7 ± 6.4
	StPT_6_	0.000	6.4	68.4 ± 6.4
	StPT_12_	0.031	6.4	68.4 ± 6.5
Adduction	StPT_0_	0.316	7.4	46.7 ± 8.9
	StPT_6_	0.339	8.0	44.3 ± 9.9
	StPT_12_	0.372	8.1	44.0 ± 10.2
External rotation	StPT_0_	0.238	8.5	56.5 ± 9.8
	StPT_6_	0.190	8.5	56.2 ± 9.4
	StPT_12_	0.240	8.8	55.7 ± 10.1
Internal rotation@90°Flex	StPT_0_	0.547	9.0	16.6 ± 16.5
	StPT_6_	0.589	8.4	21.1 ± 18.1
	StPT_12_	0.645	8.5	20.6 ± 18.6

*Note:* The good regression fits are highlighted in green.

### 3.3 Effect of Pelvic Tilt on ROM and Impingement Types

**Figure 6:**
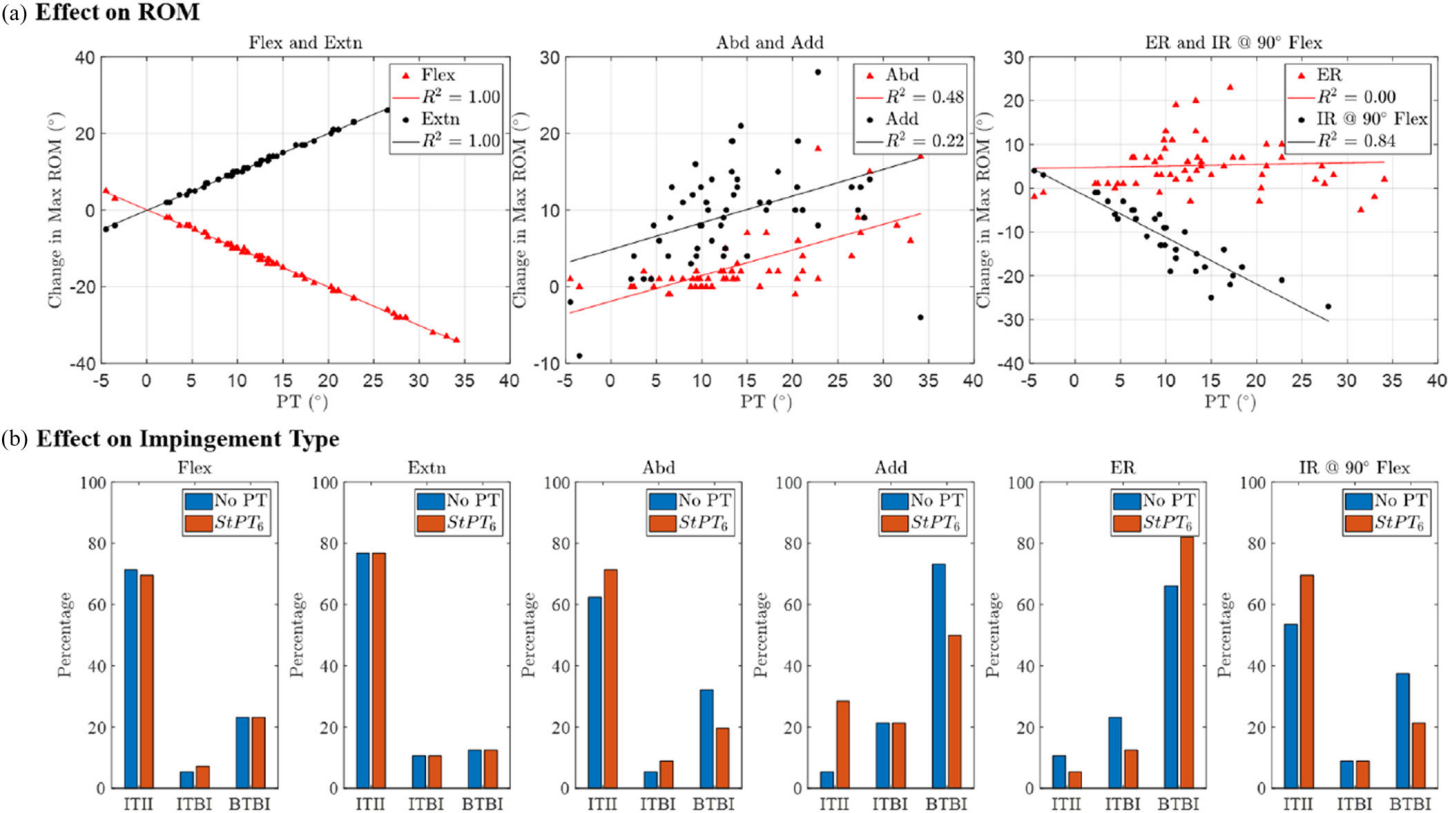
Effect of PT on maximum ROM and impingement type. (a) Change in maximum ROM for six hip motions resulting from the inclusion of StPT_6_ compared to no‐PT, and relevant correlation between change in max ROM and PT. (b) Percentage change in impingement type due to the inclusion of StPT_6_ in comparison with no‐PT scenario.

Figure 6 illustrates the change in maximum ROM between the ROM accounting for pelvic tilt at 6 months after THA (StPT_6_) and the ROM assuming no pelvic tilt. The changes in flexion and extension were perfectly correlated with PT (*R* ^2^ = 1, Figure 6a), and there was a strong correlation with IR@90°Flex (*R* ^2^ = 0.84). For every 10° change in PT, the maximum flexion/extension changed by 10.0°, and IR@90°Flex changed by 10.7°.

Upon inclusion of PT, there was no change in impingement type for flexion and extension. However, there was a higher incidence of ITII observed during abduction (8.9%), adduction (23.2%), and IR@90°Flex (16.1%) (Figure 6b). Conversely, ITII decreased by 5.4% during ER (Figure 6b). ER showed a notable increase of 16.1% in BTBI, attributed to reductions in both ITII and ITBI (Figure 6b). IR@90°Flex exhibited a 16.1% decrease in BTBI, accompanied by a corresponding increase in ITII, while ITBI remained unchanged (Figure 6b). Additionally, abduction saw a 12.5% decrease in BTBI, and adduction showed a 23.2% decrease in BTBI due to PT inclusion in ROM simulation (Figure 6b).

### 3.4 Effect of Change in Post‐THA Pelvic Tilt on ROM and Impingement Types

**Figure 7:**
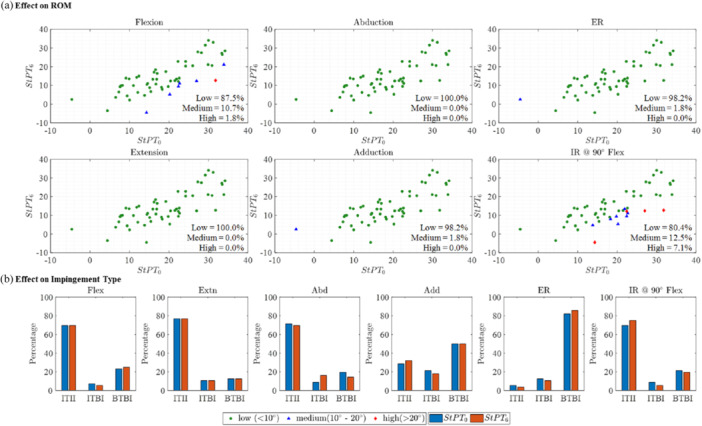
Effect of pre‐THA and post‐THA PT on maximum ROM and impingement types. (a) Different colors illustrate the amount of difference in ROM due to the change in PT from StPT_0_ to StPT_6_. Green circles represent ROM differences below 10°, blue triangles signify differences between 10° and 20°, while red diamonds indicate differences exceeding 20°. (b) Percentage of impingement type due to the use of StPT_0_–StPT_6_.

PT at 6 months after THA (StPT_6_) in standing position tilted posteriorly by 4.8° ± 6.0° in relation to the preoperative measurement (StPT_0_). A change in maximum ROM of over 10° was observed in 12.5% of patients for flexion, and in 19.6% of patients for IR@90°Flex due to the change in PT values from pre‐THA to 6 months post‐THA (Figure 7a). In contrast, no relevant changes in ROM were found for abduction (100.0%), adduction (98.2%), and ER (98.2%). In the majority of cases, no discernible change was observed in the type of impingement (< 6%) for all activities (Figure 7b).

**Figure 8:**
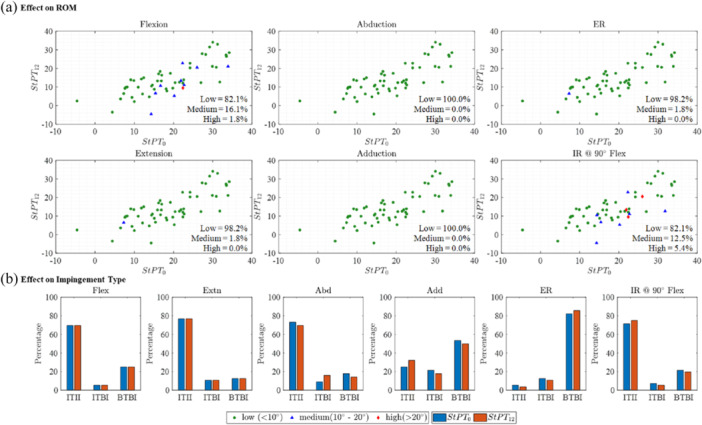
Effect of pre‐THA and post‐THA PT on maximum ROM and impingement types. (a) Different colors illustrate the amount of difference in ROM due to the change in PT from StPT_0_ to StPT_12_. Green circles represent ROM differences below 10°, blue triangles signify differences between 10° and 20°, while red diamonds indicate differences exceeding 20°. (b) Percentage of impingement type due to the use of StPT_0_–StPT_12_.

At 12 months after THA (StPT_12_), pelvic tilt in the standing position was tilted posteriorly by 4.2° ± 7.3° compared to the preoperative measurement (StPT_0_). A change in maximum ROM of over 10° was observed in 3.6% of patients for flexion and in 5.4% of patients for IR@90°Flex, attributed to the change in PT values from pre‐THA to 12 months post‐THA (Figure 8a). In contrast, no significant changes in ROM were found for abduction (100.0%), adduction (98.2%), and ER (98.2%). Additionally, in the majority of cases, no substantial change in the type of impingement was observed (< 6%) across all activities (Figure 8b).

### 3.5 Effect of Temporal Change in Pelvic Tilt Post‐THA on ROM and Impingement Types

**Figure 9:**
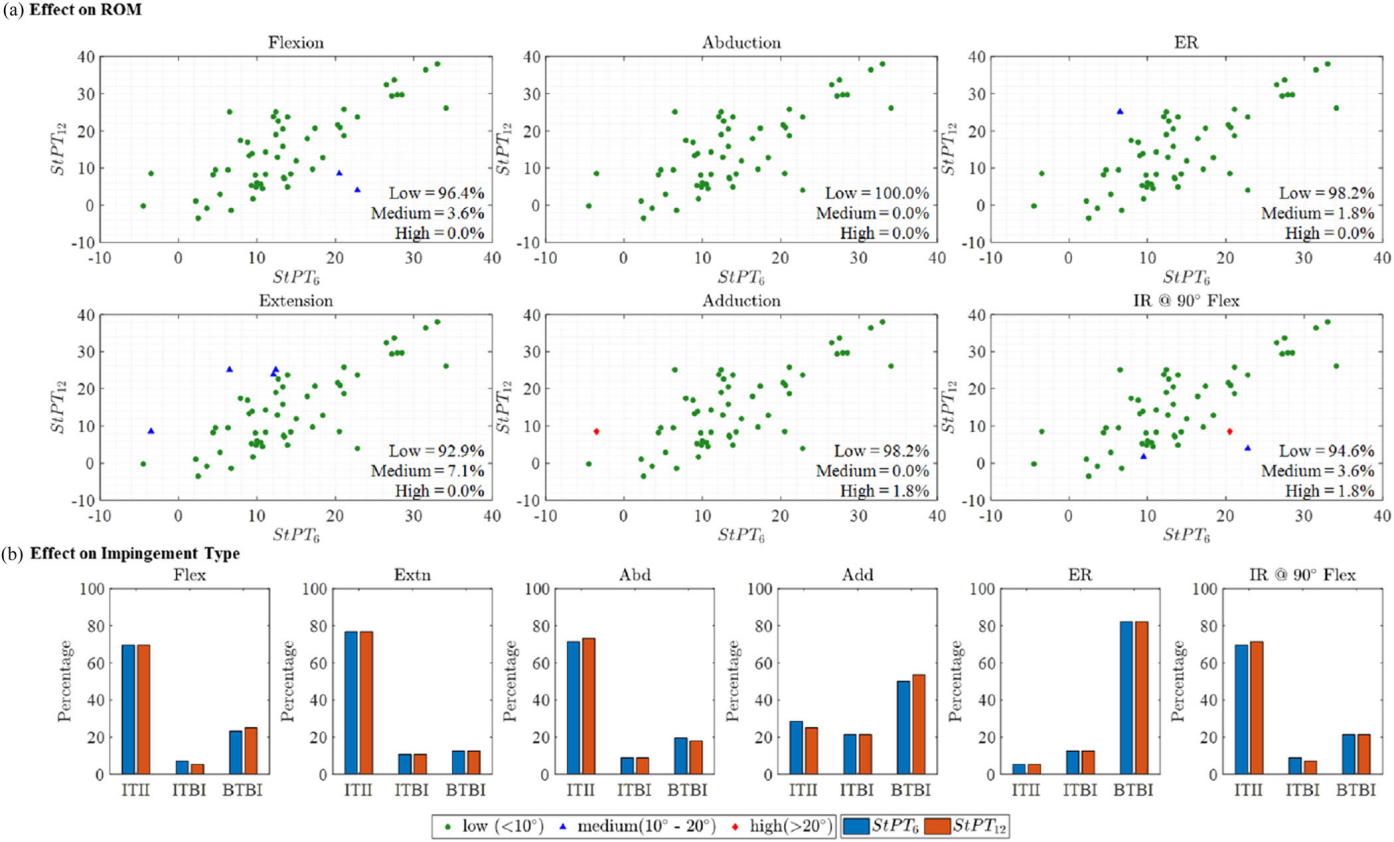
Effect of post‐THA PT on maximum ROM and impingement types. (a) Different colors illustrate the amount of difference in ROM due to the change in PT from StPT_6_ to StPT_12_. Green circles represent ROM differences below 10°, blue triangles signify differences between 10° and 20°, while red diamonds indicate differences exceeding 20°. (b) Percentage of impingement type due to the change use of StPT_6_–StPT_12_.

PT 1 year after surgery in standing position (StPT_12_) tilted anteriorly by 0.6° ± 6.9° in relation to the situation measured after 6 months (StPT_6_). Changes in ROM of over 10° were measured in 3.6% of patients for flexion, in 7.1% of patients for extension and in 5.4% of patients for IR@90°Flex (Figure 9a). In contrast, no relevant changes in ROM were found for abduction (100.0%), adduction (98.2%), and ER (98.2%). The type of impingement for all activities did not change a considerable amount (Figure 9a,b).

### 3.6 Implant Details and ITII Group

**Figure 10:**
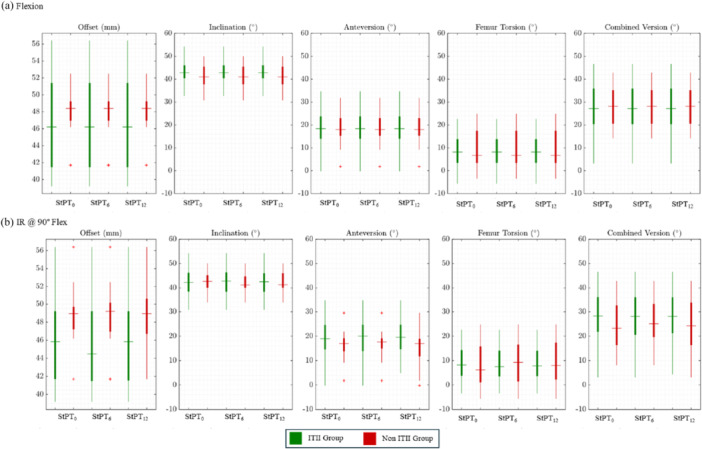
Relationship between ROM and patient characteristics, including offset, inclination, anteversion, femur torsion, and combined version, for (a) flexion and (b) IR at 90° flexion. The patient group with ROM limited due to ITII is highlighted in green, while the group with ROM limited by factors not related to ITII is shaded in red.

Figure 10 illustrates the relationship between ROMs and various patient characteristics, specifically offset reconstruction, inclination, anteversion, femur torsion, and combined version, for both flexion (Figure 10a) and IR@90°Flex (Figure 10b). The data are presented for two patient groups: one with ROM limitations attributed to ITII, and another with ROM limitations due to factors unrelated to ITII. A significant difference in offset was observed between the IR@90°Flex restricted by the ITII group (46.07 ± 4.85) and the IR@90°Flex restricted by the non‐ITII group (48.89 ± 3.38), *t*(32.15) = −2.41, *p* = 0.022, when considering StPT0. Similarly, there was a significant difference in offset between the IR@90°Flex restricted by the ITII group (45.94 ± 4.86) and the IR@90°Flex restricted by the non‐ITII group (48.67 ± 3.62), *t*(40.57) = −2.32, *p* = 0.025, when considering StPT_6_. A significant difference was also noted when considering StPT12, where the IR@90°Flex restricted by the ITII group (46.03 ± 4.83) differed from the non‐ITII group (48.64 ± 3.73), *t*(35.67) = −2.16, *p* = 0.037. However, no significant differences were observed between the two groups for other patient characteristics. It is important to note that the number of patients whose ROM was limited by ITII was twice that of those whose ROM was limited by factors unrelated to ITII.

## 4 Discussion

While there were substantial research on the evaluation of post‐THA PT [21], post‐THA ROM without PT [22, 23], and the influence of PT on healthy hip ROM [33], the relationship between PT and post‐THA ROM, as well as its association with impingement type were not thoroughly investigated. Therefore, in this study, the impact of PT and its variation on post‐THA ROM and impingement type were investigated using personalized hip motion simulation. The clinical data and patient‐specific post‐THA 3D‐CT images were used to develop the personalized hip ROM simulation for a cohort of 56 patients. Two different post‐THA PT values in standing positions, along with one pre‐THA PT value in the standing position, were utilized in this study to explore the effect of PT on post‐THA ROM: flexion, extension, abduction, adduction, ER at neutral position and IR@90°Flex. Abduction and adduction happen outside the pelvic tilt plane, unlike flexion and extension. However, pelvic tilt affects the starting position for simulating abduction and adduction. This changes the pelvic position, which might impact the ROM because the impingement point on the pelvis shifts compared to when there is no pelvic tilt. For this reason, the abduction and adduction were included in this study. The results of this study indicated that PT had a significant impact on the maximum ROM with a strong correlation between the change in ROM and the PT value for flexion, extension, and IR@90°Flex. Patients with anterior PT over 8.1° and 11.8° had a higher risk of reaching ROM boundaries regarding flexion and IR@90°Flex, respectively, required for ADL. With the inclusion of PT compared to scenarios without it, there was a notable alteration in the impingement type during abduction, adduction and IR@90°Flex, indicating a preference for higher ITII, whereas increased BTBI was observed during ER. In total, 12.5% and 19.6% of patients experienced clinically relevant ROM change for flexion and IR@90°Flex, respectively for StPT_0_–StPT_6_. However, it affected below 5.4% cases when comparing StPT_6_ and StPT_12_. Minor changes in impingement type occurred (< 6% cases) due to PT variations.

The simulated maximum post‐THA ROMs, without including PT, for flexion and IR@90°Flex, were found to be 115.2° ± 10.6° and 36.1° ± 13.9°, respectively. These values were consistent with ROM measurements reported in the literature, where flexion, extension, and abduction were 124.3° ± 14.5° and 37.5° ± 12.9°, respectively [34]. The simulated maximum post‐THA ROMs, including PT, for flexion and internal rotation at 90° of flexion were found to be 101.1° ± 16.7° and 21.1° ± 18.1°, respectively, at 6 months post‐THA. For flexion and internal rotation at 90° of flexion, the values were 100.4° ± 17.9° and 20.6° ± 18.6°, respectively, at 12 months post‐THA. The incorporation of PT into the hip joint simulation had a notable impact on both the maximum ROM and the nature of impingement, particularly for flexion, extension, and IR@90°Flex. In addition to the expected correlation of PT and flexion/extension, interestingly, a strong correlation with IR@90°Flex was also observed. An increase in anterior PT resulted in a decrease in IR@90°Flex. The vast majority of patients with anterior PT values above 10° did not meet the required ROM benchmarks for ADL regarding flexion and internal rotation. Therefore, a high anterior PT is associated with a relevant reduced flexion and IR@90°Flex, favouring anterior impingement and posterior dislocation, respectively. In the case of flexion and extension, there was no change in impingement type. However, higher rates of ITII were evident for abduction/adduction and IR@90°Flex. Additionally, bone‐to‐bone impingement becomes more prominent in cases of ER. This underscores the influence of PT on both the magnitude of ROM and the specific impingement patterns. Despite not meeting the ROM benchmark, none of the patients included in this study experienced any postsurgical complications. This might be related to the patients' soft tissue restricting clinical ROM. Furthermore, the pelvis was considered at an extreme standing PT value in this study, measured during gait performance. The lower ROM values for flexion (Figure 5a) and IR@90°Flex (Figure 5b) were generally observed for higher anterior standing PT values. However, pelvic tilt is not a static parameter but a variable one depending on the ADLs. Therefore, the pelvis can alter its position during ADLs which may help to avoid impingement even though the simulated ROM for flexion and internal rotation showed lower values for some cases, where the standing pelvic tilt was higher.

Analyzing the time‐dependent changes of PT before (StPT_0_) and after THA (StPT_6_), the pelvis tilted posteriorly by 4.8° ± 6.0°, which is consistent with the findings reported in the literature, where there was an increase in posterior PT of 4.0° ± 6.6° pre‐ and postsurgery [26]. Regarding the impact of PT on post‐THA maximum ROM, relevant changes of over 10° in ROM were found in up to 20% of patients. This effect was notable especially for flexion, and IR@90°Flex. There was also a relevant effect on extension but outside a clinically relevant area since almost all patients still reached required ROM boundaries for ADL regarding extension. This implies significant ROM changes for crucial movements postoperation in a significant number of patients. This is in line with previous studies that showed relevant changes regarding functional cup position after THA [26]. Investigating time‐dependent changes in PT after surgery, the pelvis tilted anteriorly by 0.6° ± 6.9° when comparing StPT_6_ and StPT_12_. This led to a clinically significant reduction in the ROM for 3.6% of patients for flexion and 5.4% of patients for IR@90°Flex. In contrast to the pre‐ to postoperative situation, where the ROM values increased to a relative posterior tilt of the pelvis after surgery, the pelvis tilted more anteriorly from 6 to 12 months after THA and thus decreased ROM, especially for flexion and internal rotation. Three patients experienced an increase in anterior PT after THA, resulting in reduced flexion and IR@90°Flex of over 20°, thereby increasing the risk of impingement and subsequently dislocation.

The subject‐specific ROM analysis was performed based on the intraoperative chosen position of cup and stem for each patient along with achieving successful biomechanical restoration of leg length and offset. The cup and stem were implanted according to a combined anteversion technique. Furthermore, component position as well as successful leg length and offset restoration were evaluated postoperatively [25, 35]. A strength of this study was the fact that ROM simulations were not related to one hypothetical virtual model. Instead, a cohort of THA patients with real implants was used reflecting the real position of cup and stem during surgery and variability of anatomy in real life. However, there are several limitations in the current study. First, the 3D ROM simulations focused on single ROM directions and did not account for combined motions. Second, static PT values in standing position were applied for ROM analysis. However, the pelvis is a dynamic tool, changing its position during complex activities in daily life [26]. For example, while sitting, the pelvis usually tilts backwards avoiding anterior impingement in patients with a mobile pelvis. Therefore, impingement might occur later in these cases. Finally, the effect of soft tissue has not been taken into account. Therefore, the ROM measured in this study was bony range of motion (BROM) rather than functional range of motion (FROM). However, previous studies have shown that BROM occurs for certain ranges of motion, as demonstrated in Palit et al. [28] and Han et al. [36].

In conclusion, the current study revealed a strong one to one correlation of PT and reduced flexion and IR@90°Flex. This harbors the risk of anterior impingement, and subsequently, posterior dislocation. In addition to ROM values, PT also alters the type of impingement. Furthermore, time dependent changes in PT impact postoperative ROM values. However, it has lower impact on impingement type.

## Author Contributions

**Arnab Palit:** conceptualization, formal analysis, investigation, methodology, visualization, original draft writing. **Mark A. Williams:** conceptualization, methodology, supervision, review, and editing. **Vineet Seemala:** conceptualization, formal analysis, investigation, methodology, visualization, original draft writing. **Mike Donnelly:** investigation, review, and editing. **Tobias Renkawitz:** conceptualization, investigation, methodology, supervision, review, and editing. **Markus Weber:** conceptualization, investigation, methodology, supervision, review, and editing. All authors approved the final submitted manuscript.

## Ethics Statement

This study obtained approval from the Biomedical and Scientific Research Ethics Committee (BSREC) at the University of Warwick, UK (ref: REGO‐2018‐2229 AM01 and BSREC 66/22‐23).

## Conflicts of Interest

The author Vineet Seemala was working as an intern under the WMG‐IIT KGP Student Internship Program during the initial stages of the study. The authors declare no conflicts of interest.
